# Crisis management and relational work in Swiss nursing homes during COVID-19: a qualitative multistakeholder study

**DOI:** 10.1136/bmjph-2026-005130

**Published:** 2026-10-05

**Authors:** Nathalie Heinz, Settimio Monteverde, Sophie Stephan, Andreas Plate, Stefan Neuner-Jehle, Oliver Senn, Daniel Drewniak

**Affiliations:** 1 Institute of Biomedical Ethics and History of Medicine, University of Zurich, Zurich, Switzerland; 2 Bern University of Applied Sciences, Bern, Switzerland; 3 University Hospital Zurich, Zurich, Switzerland; 4 Institute of Primary Care, University of Zurich, Zürich, Switzerland; 5 Institute of Primary Care, University Hospital Zurich, Zurich, Switzerland; 6 Institute of Primary Care, University of Zurich, Zurich, Switzerland; 7 Institute of Primary Care, University of Zurich Faculty of Medicine, Zürich, ZH, Switzerland; 8 Institute of Biomedical Ethics and History of Medicine, University of Zurich Faculty of Medicine, Zurich, Switzerland

**Keywords:** COVID-19, Qualitative Research, ethics

## Abstract

**Introduction:**

The COVID-19 pandemic confronted nursing homes across countries as they sought to prevent the spread of the virus among residents, placing substantial strain on the different stakeholder groups in healthcare. This study examines how key stakeholders of nursing homes experienced and enacted pandemic governance and how these experiences shaped organisational dynamics, relational work and preparedness for future crises.

**Methods:**

An exploratory qualitative study design was used to collect data in Switzerland between August 2024 and February 2025 across ten cantons in German-speaking and French-speaking regions. 24 semistructured, in-depth interviews were conducted with stakeholders from medicine, nursing and management as well as residents’ next of kin. Interviews were audio-recorded, transcribed verbatim and analysed using the Braun and Clarke six-phase thematic approach. The findings were interpreted using an epistemological approach and discussed with Joan Tronto’s ethics of care in order to illuminate relational and democratic dimensions of governance.

**Results:**

Three overarching themes were identified: (1) crisis governance under sustained uncertainty, (2) organisational strain and expanded relational labour and (3) selective learning and persistent gaps in preparedness. Pandemic management was characterised by the continuous local translation of rapidly changing public health directives, decentralised responsibility and the limited usefulness of formal emergency plans. Prolonged crisis conditions led to cumulative organisational strain, increased leadership pressure and heightened relational labour, particularly in communication and negotiation with next of kin. While adaptations such as infection-control workflows, vaccination logistics and testing routines improved over time, psychosocial, interpersonal and ethical dimensions remained insufficiently integrated, resulting in a fragmented process of institutional learning.

**Conclusions:**

Pandemic preparedness in nursing homes extends beyond technical readiness and formal protocols: effective crisis management depended on relational labour and the local adaptation of general guidelines. Future preparedness should integrate relational, participatory and psychosocial capacities alongside infection prevention and control measures.

**Reporting method:**

(The Consolidated criteria for Reporting Qualitative research (COREQ) checklist was followed.)

**WHAT IS ALREADY KNOWN ON THIS TOPIC**

**WHAT THIS STUDY ADDS**

- Responsibility for pandemic management was largely delegated to individual facilities without adequate resources or implementation support, with consequences for organisational functioning, staff burden and interpersonal relationships.
- Relational labour and local sense-making emerged as central but insufficiently institutionalised components of crisis management under sustained uncertainty.Psychosocial and structured ethical dimensions of care were less integrated into institutional learning on infection prevention and control.

**HOW THIS STUDY MIGHT AFFECT RESEARCH, PRACTICE OR POLICY**

- Future public health strategies for nursing homes should adopt participatory governance approaches that explicitly integrate relational, psychosocial and ethical dimensions of care.
- Emergency preparedness should extend beyond technical and procedural planning to include collective governance structures that strengthen organisational resilience in future public health crises.

## Introduction

In late December 2019, SARS-CoV-2 was discovered in Wuhan, China.^1,2^ As the seventh human coronavirus, SARS-CoV-2 spread rapidly all over the world and was declared as a pandemic by the WHO on 11 March 2020.^3,4^ Since then, the WHO has reported that COVID-19 has caused more than seven million deaths worldwide.^5^ It has become the biggest public health crisis of the past 100 years and has had significant impacts on health, social and economic systems.^6^ At the onset of the pandemic, health authorities focused on hospitals, specifically intensive care units. This prioritisation was driven by the urgent need to expand hospital capacities and prepare for triage scenarios.^7,8^ At the same time, nursing home (NH) staff, residents and next of kin (NOK) faced substantial challenges, including high infection risk, visiting restrictions, psychosocial strain and organisational disruptions within the institutions.^9–14^ NHs experienced a dual strain as they were required to respond to a rapidly evolving infectious disease while simultaneously protecting residents at particularly high risk of severe and fatal outcomes^15,16^ due to the convergence of key risk factors, including older age and comorbidities.^17,18^ In particular, elevated rates of infection and mortality were reported among residents living with Alzheimer’s disease and other forms of dementia.^19^ Residents often lived in large, densely populated settings that facilitated rapid transmission, while understaffing, insufficient access to personal protective equipment and limited staff training enabled intramural spread via care staff, thereby increasing infection risk independently of age-related vulnerability.^20^ Public health measures focused on infection prevention through non-pharmaceutical interventions that reduced physical contact, including visitation bans, movement restrictions and the suspension of social activities within NHs.^21,22^ While intended to reduce transmission, these measures were also associated with substantial unintended consequences, including increased loneliness, depressive symptoms and a decline in residents’ physical and psychosocial well-being.^23–25^ These factors, in turn, have themselves been linked to increased mortality.^26^ Restricted access equally affected NOK, limiting their ability to maintain core relationships with NH residents, provide emotional support and assist in the provision of care over extended periods.^27–29^ Consequently, NHs, positioned between health authorities, NOK and residents, increasingly became sites of ad hoc negotiations over responsibility and decision-making while continuing to manage the everyday realities of care provision. This mediation work was a key part of crisis governance, creating persistent tensions and, at times, dilemmas between the imperative to control infections and the need to minimise the detrimental effects of visiting restrictions measures on residents’ well-being and quality of life.^30–32^ Staff reported high levels of emotional exhaustion, psychological distress and moral strain. These experiences were intensified by rapid organisational changes, frequent procedural modifications and shifting decision-making responsibilities. This dynamic revealed tensions between public health directives and professional judgement and prompted the development of individual and collective coping strategies.^33–35^ The COVID-19 pandemic exposed the historical marginalisation of NHs and belatedly brought them into the public spotlight.^36^ Existing qualitative research has documented the impact of COVID-19 on NHs but has largely examined single stakeholder groups in isolation—such as staff experiences of moral strain and coping^33–35^ or NOK experiences of visitation restrictions^27,29^—leaving open how pandemic-related measures were collectively interpreted, negotiated and implemented across the stakeholder groups involved. Therefore, this qualitative study adopts a multistakeholder perspective to examine how pandemic governance was experienced and enacted and how those experiences shaped organisational dynamics, relational work and crisis preparedness.

## Materials and methods

### Study design

The study followed an exploratory qualitative approach and is part of a larger Swiss-wide research project: *COV-19-DILEMMA: Between Protection Benefit and Social Harm. Impact of Non-Pharmaceutical Protective Measures in Nursing Homes*.To obtain an overview of the pandemic measures in Swiss NHs, a nationwide online survey examined the implementation and strictness of protective measures in Swiss NHs, changes following vaccine availability, and factors associated with their adoption. Complementing this survey, the experiences and perspectives of key stakeholders from medicine, nursing and management, as well as of residents’ NOK were collected in semistructured interviews. The interviews allowed participants to raise additional aspects that had not been identified beforehand.^37^ This paper presents findings from the qualitative strand of the project.

### Setting and sample

The study was conducted across ten cantons in German-speaking and French-speaking Switzerland. NHs with different sponsorships, numbers of beds and location types (rural, suburban and urban) were included. Participants needed to fulfil the following requirements:

- Professional stakeholders (medicine, nursing, management): minimum of 6 months of work experience in a Swiss NH during the COVID-19 pandemic.
- NOK: individuals who described themselves as emotionally connected to an NH resident, regardless of biological or legal ties.

All participants were required to have the cognitive capacity to take part in the study as well as to be able to speak either German, French, Italian or English. A combination of purposive and snowball sampling was used for recruitment. In purposive sampling, participants were selected on the basis of their specific experiences with the topic being investigated. Snowball sampling was used strategically to access groups typically underrepresented in institutional research, particularly informal networks of NOK and nursing staff. Recruitment occurred via email and printed flyers. Professional stakeholders were reached through mailing lists, professional networks, and contacts who had agreed to remain in touch after a previous project. NOK were primarily reached via flyers disseminated with the help of foundations (eg, ProSenectute) and NOK groups. Flyers were available in German, French and Italian and included a QR code that opened a preformatted email to the first author so that the person can express interest easily. Alternatively, contact could be established by telephone or email. No recruitment was conducted through NH management to avoid hierarchical influence or perceived pressure. As is typical in qualitative research, the sample size could not be determined in advance.^37^ Instead, the number of interviews depended on the research question, sample heterogeneity and data richness.^38^ The achieved sample size provided sufficient information power due to the specificity of the study aim, the heterogeneity of participants and the depth of the interviews.^39^ After each interview, postscript notes were written, and an initial data saturation check was conducted after 5–7 interviews by rereading the transcripts and postscripts and writing follow-up notes. This process continued throughout data collection until no new content was identified, indicating that thematic saturation had been reached.^40,41^

### Data collection

Data were collected between August 2024 and February 2025 through 24 semistructured, in-depth individual interviews with professional stakeholders from medicine, nursing and management as well as residents’ NOK. Interviews were conducted either in person or via an online meeting platform and took place in settings chosen by participants (eg, private homes, NHs or public spaces). The research team developed two interview guides based on existing academic and grey literature—one for the professional stakeholders and one for NOK. Both guides included opening questions, probes and follow-ups, ensuring methodological consistency while allowing for narrative depth. They were initially developed in German and subsequently translated into English, French and Italian. Two doctoral researchers with expertise in qualitative methods conducted the interviews. One researcher, with a background in nursing and health sciences, conducted 20 interviews in the German-speaking region, while the second, with a background in global health, political science and international relations, conducted four interviews in the French-speaking region. All interviews were audio-recorded with the participants’ consent and stored on a secure server of the University of Zurich. Recordings were transcribed verbatim; German/Swiss German interviews (83.3%) were transcribed and standardised into High German. French interviews (16.7%) were transcribed in French, checked by the interviewer, and subsequently translated into German. Selected passages were back-translated and reviewed by native speakers to validate the translation against interpretive shifts. When an interpretive shift has been identified, the German translation was adjusted to eliminate it.

### Data analysis

Data were analysed using Braun and Clarke’s (2006) thematic analysis approach. The qualitative data were managed and analysed with MAXQDA (V.24.11.0; VERBI Software, Berlin, Germany). An initial codebook was developed through a combined deductive–inductive process and discussed with project members. This approach enabled the integration of content informed by both interview guides and through inductive coding of the interview material, allowing new codes to emerge from the multistakeholder perspectives. The study followed an interpretivist epistemological stance, acknowledging that participants’ accounts reflect their situated experiences, perspectives and meanings. Accordingly, the analysis remained closely oriented toward how participants described their own realities. The findings were interpreted through the lens of Joan Tronto’s ethics of care.^42,43^ This framework enabled us to examine how responsibilities, relational dynamics and institutional boundaries shaped care practices and governance processes in Swiss NHs during the pandemic. An overview of the six steps of the analytic process is provided in table 1. To enhance transparency, the research team applied Braun and Clarke’s 15-item checklist for evaluating thematic analysis and adhered to the Consolidated criteria for Reporting Qualitative research (COREQ) reporting guidelines.

**Table 1:** Phases of thematic analysis

Phase	Description of the process
1 Familiarisation	The analysis started with data familiarisation. One researcher (NH) carefully read each transcript and postscript several times and summarised each interview with memos and topics in a mind map. Initial impressions were then discussed within the research team
2 Generating codes	The first author (NH) coded the dataset and regularly met with two senior researchers (DD/SM). The coded data were examined to understand how different codes might be combined to identify patterns of broader meaning (ie, potential themes).
3 Constructing themes	The process of developing initial themes by using the codes was conducted by the smaller research team (SM/DD/NH).
4 Revising themes	All themes were continuously reviewed and further developed regarding (a) each individual’s data set and (b) the overall data set (regarding commonalities and differences within themes).
5 Defining themes	The entire research group discussed the themes. The resulting feedback led to the refinement of the themes into a final pattern, thereby answering the corresponding research question.
6 Producing the report	Finally, the first author (NH) wrote the study report, which was critically reviewed by all co-authors.

### Trustworthiness and methodological rigour

Several strategies were employed to enhance the trustworthiness and methodological rigour of the study. The semistructured interview guides were developed within the research team, who come from interdisciplinary backgrounds (ethics, medicine, psychology, nursing and health science) and reviewed by experts in qualitative methodology. They were pilot-tested with representatives from nursing, medicine and NOK using think-aloud techniques^44^ to ensure clarity, relevance and contextual appropriateness.^45^ Throughout data collection and analysis, regular meetings were held within the research team to critically discuss emerging findings, refine the coding framework and reflect on analytic decisions. In addition, the interdisciplinary peer mentoring group *QualiZüri* provided ongoing methodological support for qualitative research. Reflexivity was actively integrated into the research process through field notes and structured reflection sessions.^46^ These practices enhanced transparency, traceability and critical examination of the researchers’ roles, assumptions and potential biases.^47^ Preliminary findings were discussed within the core research team and in an interdisciplinary research colloquium. Feedback from these exchanges informed further refinement of the thematic structure and strengthened the analytical interpretation.

## Results

### Participant characteristics

Three individuals declined participation at the beginning of recruitment. One person withdrew due to discomfort with audio recording and two did not respond to follow-up contact. In total, 24 semistructured in-depth interviews were conducted with professional stakeholders, including nursing staff (33.3%), management staff (20.8%) and general practitioners (GPs) (16.7%) as well as with NOK (29.2%). Interviews had a mean duration of 52 min (range 32–88 min) and were conducted either in person (62.5%) or via an online platform (37.5%). Of the participants, 62.5% were female. An overview of participant characteristics is provided in tables 2 and 3.

**Table 2:** Characteristics of (bereaved) next of kin and (deceased) residents in nursing homes

Category	Subcategory	Number of NOK (total: 7)	%
Age of next of kin, years (M±SD)	–	–	60.9±8.3
Gender of next of kin	Female	6	85.7
	Male	1	14.3
Relationship to the resident in NH	Children	6	85.7
	Grandchildren	1	14.3
Professional background	Social/healthcare services	5	71.4
Visit frequency before March 2020	Daily	3	42.9
	2–3×/week	1	14.3
	1–2×/week	2	28.6
	Once/week	1	14.3
Age of (deceased) residents, years (M±SD)	–	–	91.1±4.5
Gender of (deceased) residents	Female	6	85.7
	Male	1	14.3
Room type	Single room	7	100
Time of death	4th quarter 2020	4	57.1
	1st quarter 2024	1	14.3
	2nd quarter 2024	1	14.3
	Alive at data collection	1	14.3
Facility type*	Public	2	33.3
	Private	4	66.7
Facility region	Urban	4	57.1
	Suburban	2	28.6
	Rural	1	14.3

Percentages are based on n=7 unless otherwise indicated.

* Facility type missing for one case; percentages based on valid responses (n=6).

M, mean; NH, nursing home; NOK, next of kin.

**Table 3:** Characteristics of professional stakeholders in nursing homes

Category	Subcategory	Number of stakeholders (total: 17)	%
Age, years (M±SD)*	–	–	52.8±8.6
Gender	Female	9	52.9
	Male	8	47.1
Position	Nursing home manager	5	29.4
	General practitioner	4	23.5
	Nurse	8	47.1
Employment status	Currently employed	16	94.1
	Retired	1	5.9
Position since before March 2020†	Same position	12	70.6
	Position change	5	29.4
	Position change: facility change	4	23.5
	Position change: retired since 2020	1	5.9
Facility type‡	Public	6	40.0
	Private	9	60.0
Facility region	Urban	7	41.2
	Suburban	2	11.8
	Rural	8	47.1
Facility size	≤50 residents	4	23.5
	51–100 residents	4	23.5
	101–200 residents	9	52.9

Percentages are based on n=17 unless otherwise indicated. Percentages may not sum to 100 owing to rounding*.*

* Age not reported by two participants (n=15).

† ‘Position change: facility change’ and ‘position change: retired since 2020’ are subsets of ‘position change’ and are not additive with the main categories.

‡ Facility type missing for two participants; percentages based on valid responses.

M, mean.

### Core themes

Three overarching themes, comprising eight subthemes, were identified, capturing how professional stakeholders and NOK experienced, interpreted and retrospectively made sense of pandemic management in Swiss NHs: (1) crisis governance under sustained uncertainty; (2) organisational strain and expanded relational labour and (3) selective learning and persistent gaps in preparedness (figure 1; additional supporting quotes are provided in the online supplemental material). Across accounts, pandemic management was described as an ongoing process rather than a temporary emergency, shaped by continuous uncertainty, repeated adjustments and everyday compromise between institutional requirements and care realities. The themes are interrelated: governance under sustained uncertainty produced cumulative organisational strain and relational labour, which in turn shaped what lessons were—and were not—learnt to inform future preparedness. Bringing professional and NOK perspectives together made visible not only shared challenges but also points of tension and misalignment between stakeholder groups. The rest of this section presents each theme and its constituent subthemes.

**Figure 1:**
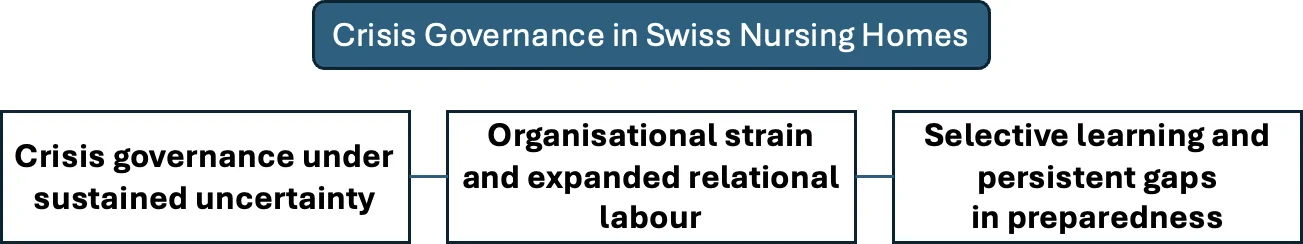
Overview of the findings.

#### Theme 1: crisis governance under sustained uncertainty

Across all stakeholder groups, pandemic management in NHs was described not as discrete emergency phase but rather as an ongoing state of uncertainty that permeated everyday decision-making. This theme comprises three subthemes: (1.1) rapidly changing rules issued with little advance notice (1.2) increased mediation work in translating non-context-specific guidelines into practice and (1.3) high internal decision-making responsibility and unclear roles.

##### Rapidly changing rules with limited lead time

Participants emphasised that uncertainty did not diminish after the initial outbreak phase but remained a defining feature throughout successive pandemic waves. Decisions and procedures were perceived as unstable over time, with guidance changing faster than it could be consolidated in practice. As one GP noted,

What we had defined on Monday was already wrong by Thursday. (GP1, Pos. 55)

Formal preparedness plans, where they existed, were therefore widely perceived as offering limited practical guidance once external regulations began to change rapidly and unpredictably.

##### Increased mediation work: implementing non-context-specific guidelines into practice

Under these conditions, NH administrators and GPs described pandemic governance as a process of continuous translation. Public health directives were often experienced as abstract, incomplete or frequently revised, requiring rapid interpretation and contextual adaptation within each institution. This translation work had to be carried out within very short time frames and with limited external support. As one NH administrator explained,

On Friday evenings, we regularly ended up reorganising the nursing home again so that the regulations would be met by Monday morning. (NHM3, Pos. 5)

For nursing staff, this mediation work was primarily experienced through intensified implementation pressure in everyday care practices. Top-down regulations required immediate enactment even when they conflicted with established practices and relational care needs. This tension was particularly evident in dementia care, where strict infection control measures were perceived as incompatible with residents’ behavioural and relational requirements. Nursing staff described relying on situational judgement, informal negotiation and pragmatic adaptation to reconcile formal directives with care realities. As one member of the nursing staff stated,

You just tried somehow to implement the instructions that came from above with whatever measures you could, knowing that it wouldn’t work. Especially in dementia care, it just doesn’t work. (N1, Pos. 13)

##### High internal decision-making responsibility and unclear roles

Decision-making responsibility was perceived to have shifted from the Federal Office of Public Health (FOPH) to NHs themselves, leaving NHs with a heightened sense of responsibility while operating within role definitions and governance structures that were often unclear or inconsistently communicated. At the same time, the demands on NH administrators increased as they took on additional operational and front-line tasks, which further constrained strategic oversight. One administrator reflected on this change, noting that

The overview from a bird’s-eye perspective was missing. Someone who holds the threads together and manages the organisational aspects drops out the moment you are running around at the frontline yourself. (NHM2, Pos. 59)

GPs similarly reported expanded responsibilities and unclear role boundaries, resulting in a pragmatic redistribution of tasks that emerged informally rather than through formal clarification.

Overall, theme 1 demonstrates that pandemic governance in NHs was not stabilised through preparedness plans but emerged through continuous local sense-making: rapidly changing external rules (1.1) necessitated ongoing mediation work within facilities (1.2), which in turn concentrated responsibility on institutions and individuals whose roles remained ambiguous (1.3). These governance conditions formed the backdrop against which organisational strain and relational labour intensified over time.

### Theme 2: organisational strain and expanded relational labour

Sustained uncertainty translated into cumulative organisational strain that extended far beyond periods of acute infection risk. This theme comprises two subthemes: (2.1) workforce strain and (2.2) relational labour as the invisible infrastructure of crisis management.

#### Workforce strain

Across professional groups, participants described the pandemic as a prolonged crisis that progressively eroded organisational reserves rather than stabilising them. Frequently changing regulations required continuous adjustments to routines, workflows, and responsibilities, contributing to persistent pressure on an already overstretched workforce. This pressure included, beyond the challenge of practical implementation, sustained physical and emotional strain on staff. As one member of the nursing staff recalled,

Just keep going. That was my motto. It was exhausting […] After the pandemic came exhaustion. Even during the pandemic […] The exhaustion was definitely both physical and mental. (N3, Pos. 61–67)

Leadership roles were particularly strained under these conditions. Administrators and GPs described compressed role expectations that combined regulatory implementation, operational coordination, staff support and conflict management. Implementing new or revised measures often required substantial organisational effort, frequently under time pressure and with limited external guidance. As one GP noted,

Whenever new official regulations came out, this required enormous organisational effort. They often came in rapid succession, with very little lead time, and teams were left alone with the implementation*.* (GP4, Pos. 26)

As a result, leadership work increasingly shifted from strategic planning to continuous mediation and problem-solving.

#### Relational labour as invisible infrastructure of crisis management

A central feature of organisational strain was the expansion of relational labour. Managing pandemic-related measures required constant communication, explanation and negotiation to maintain cooperation and adherence among staff, residents, and externals. This work was described as cyclical rather than episodic, since changing rules repeatedly had to be reintroduced and reinforced. One NH administrator explained,

When things started up again, it required renewed effort to ensure that people adhered to the rules; hand hygiene, wearing masks, and so on. This involved residents, staff, and visitors alike and required repeated communication and coordination. In the end, it was again a substantial effort*.* (NHM3, Pos. 13)

Although widely recognised as necessary for maintaining day-to-day functioning, this relational work remained largely invisible in standard operating procedures and, importantly, formal workload calculations. In addition to communication demands, participants described situational expansions of professional responsibility that compensated for gaps in care provision. GPs reported informally taking over responsibilities beyond their usual scope in order to maintain continuity of care. As one GP stated,

I basically took over the care of patients in the nursing home for another general practitioner as well*.* (GP3, Pos. 2)

Such arrangements were framed as pragmatic responses to extraordinary circumstances but further concentrated responsibility on individual actors rather than delegating it through formal organisational structures. Leadership was also described as acting as a buffer between external mandates and frontline realities. Participants reported deliberately filtering, translating, or delaying information to protect staff from the cumulative burden of constant change. As one GP reflected,

What was considered right yesterday was already nonsense the next day. And that might be acceptable at an inner-circle level, but I couldn’t pass this on to staff who were dealing with residents in their daily work*.* (GP3, Pos. 30)

While this buffering function was perceived as essential for team management, it also intensified emotional and organisational demands on leaders.

Overall, theme 2 shows that prolonged crisis management in NHs relied heavily on expanded relational labour as a stabilising force: the strain on staff and leadership (2.1) was absorbed largely through communication, mediation, and informally taking responsibility (2.2). This enabled continuity of care under sustained uncertainty but simultaneously shifted responsibility onto individuals and informal practices, making organisational resilience dependent on personal endurance rather than institutional capacity.

### Theme 3: selective learning and persistent gaps in preparedness

Over the course of the pandemic, NHs implemented a range of adaptations aimed at stabilising operations and reducing uncertainty. However, learning was described as uneven across domains. This theme comprises three subthemes: (3.1) the establishment of new workflows and procedural learning over time, (3.2) the absence of structured psychosocial processing, ethical deliberation and reflection (3.3) the exclusion of NOK and the prevalence of one-way communication.

#### Establishment of new workflows and procedural learning over time

Across professional groups, participants described gradual improvements primarily within technical and procedural domains, including testing routines, cohorting practices, vaccination logistics, and infection control workflows (c.f. table 4). With recurring exposure to outbreaks, these operational aspects became increasingly routine and predictable, contributing to greater confidence in managing acute situations. The emergence of vaccines was widely described as a turning point, as one administrator put it, it was “a huge relief, because it was very quickly associated with easing of restrictions” (NHM4, Pos. 57). Once vaccination campaigns had been established, planning became more reliable and day-to-day crisis management less volatile. These developments reinforced perceptions that certain dimensions of pandemic management could be effectively addressed through technical solutions and procedural refinement.

**Table 4:** Professional stakeholders’ perspectives on lessons learnt and perceived gaps in pandemic preparedness

Domain	Implemented adaptations	Perceived gaps	Illustrative quote
Material and logistics	Expansion and stockpiling of protective equipment	Preparedness framed primarily as stockpiling; strategic use and distribution less considered	Better, by having enough protective equipment available. (N3, Pos. 55)
Testing and monitoring	Establishment of testing and isolation routines	Frequently revised testing and isolation requirements demanded short-notice decisions	A lot of decisions had to be made at very short notice. (GP4, Pos. 10)
Spatial organisation	Cohorting, creation of separate units or wards	Structural limitations not allowing implementation everywhere	Residents who were ill could stay in this large room, separate from the others (…) I would say that is almost a must-have. (NHM1, Pos. 45)
Digital communication	Use of tablets and video calls	Inability to replace physical proximity and relationships	Just to establish any kind of contact at all. (N3, Pos. 19)
Visitation policies	Partial easing of visitation restrictions	Blanket bans, lack of differentiation	Such a visitation ban is simply not acceptable. (N1, Pos. 57)
End-of-life care	Exceptions granted at the end of life	Restrictive implementation, lack of dignity	Not a good basis for end-of-life care. (GP4, Pos. 12)
Psychosocial support	Informal peer exchange	Absence of structured psychosocial support	Or you meet in the stairwell and say: 'I need someone to talk to.’ Otherwise, you get caught in this spiral of self-doubt. (N8, Pos. 106)
Reflection and processing	Individual reflection (no internal follow-up)	Lack of institutional follow-up and collective processing	That we never got answers to these issues. (GP3, Pos. 28)

Domains are ordered following the subtheme structure of theme 3, moving from technical-procedural domains (cf. subtheme 3.1) to psychosocial and ethical domains (cf. subtheme 3.2).

#### Absence of structured psychosocial processing, ethical deliberation and reflection

In contrast, learning related to psychosocial, ethical, and relational dimensions of care was described as uneven or mostly absent. Across stakeholder groups, these dimensions were sidelined in favour of infection prevention and control strategies (c.f. table 4). Psychosocial concerns were typically dealt with informally, through individual coping or ad hoc peer exchange, without being embedded in organisational decision-making structures. Similarly, infection control decisions proceeded without structured ethical deliberation, so that the relational conflicts they generated—illustrated, for example, by the tensions in dementia care described in theme 1—had to be resolved situationally by individual staff members. One GP articulated the resulting unmet need explicitly:

Nursing staff need spaces to address their dilemmas*.* (GP4, Pos. 46)

Staff and administrators noted a corresponding lack of structured opportunities for collective reflection, debriefing or systematic sense-making once acute crisis phases subsided. Experiences of moral strain, emotional exhaustion, and unresolved ethical tensions were rarely translated into formal organisational processes, limiting the integration of lived experience into sustained preparedness strategies. While professional stakeholders primarily experienced this gap as an absence of reflective and supportive structures within their organisations, NOK described its consequences as unprocessed personal distress persisting long after the acute crisis. As one NOK recounted,

Even today, I still struggle to take the tram along that route… it is difficult for me. (NOK3, Pos. 106)

#### Exclusion of NOK and prevalence of one-way communication

This gap was particularly evident from the perspective of NOK (c.f. table 5). They reported that despite being profoundly affected by visitation restrictions and organisational measures, they were not invited to participate in post-crisis reflection. As one NOK stated,

**Table 5:** Next-of-kin perspectives’ on lessons learnt and recommendations for future preparedness

Domain	Problems experienced during the pandemic	Proposed improvements/learning needs	Illustrative quote
Autonomy and participation	Decisions made over residents’ heads	Involvement of residents and NOK in risk assessment and decision-making	“Why doesn’t anyone ask me what I want?” (NOK7, Pos. 41)
Visitation policies	Blanket visitation bans, lack of differentiation	Avoidance of complete bans; flexible, individualised visitation models	“Visits were extremely restrictive; they were simply stopped.” (NOK1, Pos. 37)
Differentiated protection concepts	Uniform rules for very different needs	Grouping according to risk preferences (eg, protection vs social closeness)	“Those who want social contact should be allowed to have it.” (NOK1, Pos. 55)
Spatial and visual solutions	Lack of orientation and choice	Visual indicators (eg, coloured signs on doors) to signal visitation preferences	“A green or red heart on the door(…)then you know who wants visitors.” (NOK4, Pos. 119–127)
Social participation	Isolation, loneliness, loss of meaning	Protecting social contact as a basic need, even in crises	“That was her lifeline.” (NOK1, Pos. 5)
End-of-life care and farewell	No farewell, restrictive visiting hours, absence of rituals	Dignified end-of-life care with presence of NOK	“No farewell, nothing.” (NOK6, Pos. 113)
Psychosocial impact on NOK	Powerlessness, helplessness, long-term distress	Opportunities for processing and post-crisis support	“I’m glad I can talk about it here.” (NOK3, Pos. 96), “It was basically a trauma, a catastrophe.” (NOK4, Pos. 5)
Consistency and transparency of rules	Inconsistent implementation of rules across facilities	Transparent communication and clear justification of measures	“It was completely different from one nursing home to another.” (NOK2, Pos. 75)
Societal embeddedness of NHs	NHs as closed-off institutions	Stronger societal integration, intergenerational concepts	“Nursing homes should remain part of society.” (NOK1, Pos. 73)
Quality of life vs life prolongation	Protection prioritised at all costs	Prioritising quality of life and individual values	“It cannot be the goal to live as long as possible at any price.” (NOK1, Pos. 57)

Domains reflect problems experienced by NOK during the pandemic and the improvements they proposed for future crises (cf. subthemes 3.2 and 3.3).

NH, nursing home; NOK, next of kin.

No, there was no follow-up or reflection at all […] We, as next of kin were never involved. (NOK4, Pos. 135)

Consequently, experiential knowledge held by NOK remained largely external to institutional learning processes. Several NOK further described that while communication mechanisms were in place, they were predominantly administrative and informational rather than relational or participatory. As one participant explained,

Often, communication from the administration came in the form of a letter (…) But we never heard anything about how the residents were actually doing (…)*.* (NOK7, Pos. 30)

From this perspective, improvements in communication infrastructure did not translate into meaningful dialogue or inclusion. Overall, theme 3 demonstrates that institutional learning from the COVID-19 pandemic in NHs remained incomplete: while technical and procedural adaptations became routinised over time (3.1), the psychosocial and ethical dimensions of care were treated as subsidiary concerns rather than being integrated into preparedness structures (3.2), and the experiential knowledge of NOK remained excluded from institutional learning processes (3.3). As a result, preparedness was predominantly framed in terms of tangible resources, protocols and technical capacities, rendering learning selective and potentially fragile. Tables 4 and 5 synthesise these learning patterns across governance dimensions, contrasting implemented adaptations—predominantly technical—with persistent gaps in psychosocial processing and participation.

## Discussion

### Summary of main findings

This qualitative study examined how professional stakeholders and residents’ NOK experienced and enacted pandemic governance in Swiss NHs. Across stakeholder groups, pandemic management was described as a prolonged period of sustained uncertainty in which responsibility for implementing rapidly changing directives shifted to individual facilities, while formal emergency plans offered limited practical guidance. Maintaining care provision relied on cumulative relational labour and informal redistributions of responsibility. While technical and procedural elements of infection control became consolidated over time, psychosocial, interpersonal and ethical dimensions of care were less consistently integrated into institutional learning and preparedness.

#### Interpreting the findings through the lens of Tronto’s ethics of care

As outlined in the methods section, the findings were interpreted through Tronto’s ethics of care.^42,43^ We considered this framework particularly appropriate because it conceptualises care as a political and institutional practice—distinct from an exclusively private or interpersonal morality—linking ethics, institutional arrangements and democratic participation. It therefore offers a suitable lens for connecting stakeholders’ experiences with the governance-level implications of our findings. Tronto describes care as a process unfolding across five phases (caring about, taking care of, care-giving, care-receiving and caring with), each associated with an ethical quality (attentiveness, responsibility, competence, responsiveness and solidarity, respectively).^42,43^ These phases provide a structured way to analyse how responsibilities are allocated, how relational dynamics evolve and how institutional boundaries shape care practices.

Regarding the governance conditions described in theme 1, the findings indicate that the responsibility for pandemic management was largely delegated to individual NHs, which faced a previously unknown situation. Public health directives were formulated in general terms, and facilities had to translate these into local practice on their own. The necessary resources—such as personal protective equipment, adequate staffing, implementation support and guidance—were frequently missing, which pushed management to their limit. From a care-ethical perspective, this constellation reflects a tension that Tronto identifies in the allocation of care responsibilities: responsibility for the care of residents was delegated downward within the organisational hierarchy, while the resources and conditions required for competent care giving were not adequately provided.^43^ The expanded relational labour captured in theme 2—communication, mediation, and emotional support—was central to everyday functioning, yet remained informal and institutionally under-recognised, which is symptomatic of the invisibility of care work that Tronto identifies as a structural challenge of contemporary care arrangements.^43^ The limits of this governance approach became particularly evident in the role assigned to NOK, as discussed in theme 3. Despite their contribution to residents’ well-being, they were frequently treated as external risk factors at the beginning of the pandemic and were often only marginally involved in decision-making processes. From the perspective of ‘caring with’, which understands care as a shared and democratic responsibility, this exclusion represents a clear deficit in participation.^42^ Where trust, involvement and shared learning are limited, experiences of relational loss and moral strain can hardly be integrated into future preparedness.

#### Changes across pandemic phases

Across stakeholder groups, participants recalled the early phase of the pandemic in vivid detail, frequently referring to specific dates, sequences of events and key moments of realising what was happening. During this period, as noted above, NOK tended to be treated as risk factors following the logic of official pandemic governance rather than as resources for effective pandemic management^48^—an in-group/out-group dynamic that may have undermined trust and inclusive learning. While technical and procedural domains such as cohorting and testing routines improved over time, psychosocial support and structured ethical reflection were less consistently integrated into institutional learning and preparedness, and improvements were perceived as fragile once novel regulations or acute pressures subsided. Some of these dynamics are closely tied to the Swiss context—particularly the federalist division of responsibilities across federal, cantonal and municipal levels, which amplified local implementation work and interinstitutional variation. For this reason, the new Swiss pandemic plan^49^ proposes that collaboration among all actors involved must be strengthened to ensure coordination in the event of a pandemic. The FOPH and the actors involved are therefore required to continuously maintain structures and mechanisms for coordination and consultation and to communicate transparently with one another; actors are expected to prepare their tasks and responsibilities and to demonstrate a willingness to practise regularly. Internationally, similar patterns and effects have been reported, including persistent moral dilemmas, relational disruptions and long-term psychosocial strain among residents, families and care staff.^20,24,27,30^ Calls for trauma-informed and co-designed approaches to post-pandemic care therefore align the perspectives of the ethics of care emphasising shared responsibility, trust and democratic participation.^42,50^

#### Lessons learnt for improving pandemic preparedness

Our findings highlight three interrelated gaps in preparedness as experienced by stakeholders: the absence of structured psychosocial and reflective support, the exclusion of NOK from decision-making and post-crisis learning and the lack of implementation support for facilities translating rapidly changing directives (tables 4,5). Taken together, these gaps reveal how relational, participatory and institutional dimensions of care were insufficiently integrated into preparedness structures. Drawing on the ethics of care and work on participatory governance in long-term care, we propose three directions for addressing these gaps:

First, participatory structures such as resident and NOK councils should be established outside crisis periods, so that ‘caring with’ is institutionally anchored before the next emergency, and the status of NOK is appropriately recognised—not as risk factors or mere visitors, but as explicit partners in care and governance. This aligns with expert recommendations from a federally commissioned Swiss study^51^ and with models of patient and public involvement, such as the Montreal Model,^52^ which are increasingly being integrated into Swiss healthcare quality frameworks.^53^

Second, coordination and implementation support between federal, cantonal and municipal authorities and NHs should be strengthened, ensuring that facility leadership is not left alone to interpret and enact protective measures. The documented variation in restrictiveness across Swiss facilities and language regions underscores the need for transparency and shared guidance.^51^

Third, psychosocial support and structured ethical reflection should be embedded in preparedness planning, so that moral distress and relational strain are addressed proactively rather than reactively. This echoes calls to integrate psychosocial and ethical dimensions in national pandemic plans^54^ and reflects care-ethical perspectives that consider relational, participatory and psychosocial capacities as core components of preparedness rather than optional add-ons.^42,43^ The revised Swiss national pandemic plan, published in 2025 as a living document^49^ to be continuously updated, provides a timely opportunity to incorporate these dimensions: By embedding participatory governance, coordination mechanisms and psychosocial support into preparedness planning, Switzerland and other countries can move toward a more ethically robust and resilient pandemic response.

### Limitations

Several limitations should be considered when interpreting the findings. First, although recruitment extended to the Italian-speaking region, no individuals or institutions from the canton of Ticino responded to the study invitation, limiting complete national coverage. This is noteworthy given that Ticino was among the first regions in Switzerland to be affected by COVID-19, owing to its proximity to severely affected Northern Italy,^55^ and implemented comparatively strict pandemic measures.^51^ Criminal proceedings against NH leadership concerning pandemic management in Ticino^56^ may explain reservations to participate. Second, residents were not interviewed directly, as the study focused on governance and crisis management processes and many residents who had experienced the pandemic in NHs had since died or were experiencing cognitive decline; their perspective is documented in a federally commissioned Swiss study conducted closer to the events.^51^ Third, interviews were conducted in German/Swiss German and French between August 2024 and February 2025, more than a year after the WHO declared the end of the COVID-19 global-health emergency. Retrospective accounts may therefore be subject to recall bias. Fourth, interviews were translated into High German; despite spot back-translation and native-speaker review, translation might have introduced minor nuances that could have influenced interpretation. Several participants described the interviews as emotionally meaningful and expressed appreciation for the opportunity to reflect on their experiences and contribute to collective learning, despite the emotional strain associated with revisiting painful memories. Given the emotional burden described during the interviews, the research team decided not to re-contact participants for member checking, which may have impacted the findings.

## Conclusions

This study examined how professional stakeholders and residents’ NOK experienced pandemic governance in Swiss NHs, identifying three interrelated themes: crisis governance under sustained uncertainty, organisational strain and expanded relational labour and selective learning with persistent gaps in preparedness. Pandemic responses relied on informal coordination and extensive relational work. While NHs demonstrated considerable adaptive capacity, preparedness remained largely technical and procedural.

Drawing on Tronto’s ethics of care, we argue that psychosocial, relational and ethical dimensions are not peripheral but constitutive of effective crisis governance. Tronto’s framework highlights how care unfolds through phases of responsibility, competence, responsiveness and solidarity, and how these must be institutionally supported rather than left implicit. NHs should therefore be recognised not merely as sites of infection control but as relational and deliberative care settings—living spaces where people dwell as distinct yet interconnected social beings. Strengthening preparedness therefore calls for integrating participatory governance, sustained workforce support and psychosocial capacities alongside infection prevention and control. Further research is needed to operationalise this reconceptualisation and to explore how care-ethical commitments, especially the democratic dimension of ‘caring with’, can be embedded across governance levels.

## Supplementary material

10.1136/bmjph-2026-005130 online supplemental file 1

## Data Availability

Data are available on reasonable request.
