# Supplementary material for: Crisis management and relational work in Swiss nursing homes during COVID-19: a qualitative multistakeholder study
Source: BMJ Public Health. 2026 Oct 5;4(4):e005130. doi: 10.1136/bmjph-2026-005130 (PMC13646948; doi:10.1136/bmjph-2026-005130)
Supplement: Supplementary file 1 [file bmjph-4-4-s001.pdf]

Additional supporting quotes

Theme	Subtheme	Quotes
**Crisis governance under sustained uncertainty**	Rapidly changing rules with limited lead time	"[...] that visits were suddenly no longer allowed, from one day to the next." (NOK2, Pos. 3)<br><br>"[...] they came in very rapid succession and were repeatedly revised. From my perspective, they were often poorly timed, with very little lead time." (GP4, Pos. 26)
	Increased mediation work: implementing non-context-specific guidelines into practice	"And then the teams were often not supported in the implementation, right? They were not professionally supported. They were simply given instructions on how things had to be done." (GP4, Pos. 26)<br><br>"We strictly followed the rules and guidelines, although we realized that sometimes it was practically impossible to do so, but it was not our intention to disregard them." (NHM5, Pos. 125)
	High internal decision-making responsibility and unclear roles	"[...] that from then on, as the nursing home physician, I would take responsibility for COVID. And that I would also take over testing for patients I was not formally responsible for." (GP1, Pos. 11)<br><br>"During the pandemic, I had several different roles." (N3, Pos. 7)

**Organisational strain and expanded relational labour**	Workforce strain	"So, the level of fear was incredible [...] There were two or three staff members who were effectively no longer functional. Actually, they should have been sent home. They were completely paralysed; they no longer knew what to do." (GP1, Pos. 13)<br><br>"No matter whether I had two or three days off, in the end I still felt as if I had only been home for half an hour." (N8, Pos. 100)
	Relational labour as invisible infrastructure of crisis management	"I still remember that in March I worked 25 days in a row, simply because there was an enormous need for communication." (NHM4, Pos. 3)<br><br>"The challenge was to remain as calm as possible [...] to spread calm, to be present and to make sure that we looked after one another." (NHM1, Pos. 63)
**Selective learning and persistent gaps in preparedness**	Establishment of new workflows and procedural learning over time	"We started developing the first emergency concepts because we realised: oops, we have certain gaps. I would say staffing, care, cleaning, and the emergency kit are the main pillars." (NHM2, Pos. 41)<br><br>"Later on - although we did not know this at the time - we realised that we could actually be quite successful with relatively few hygiene measures." (GP1, Pos. 21)

	Absence of structured psychosocial processing, ethical deliberation, and reflection	"For almost two years, I could hardly talk about it without crying." (N1, Pos. 101)<br>"I believe solutions should be found so that people are better supported psychosocially - staff, and certainly residents as well." (NHM3, Pos. 51)
	Exclusion of next of kin and prevalence of one-way communication	"(...) a complete severing of ties with the outside world." (NOK7, Pos. 2)<br><br>"Next of kin were, to some extent, excluded." (NHM4, Pos. 49)<br><br>"[...] would be more careful today about this deprivation of relatives [...] Not a blanket approach of closing all nursing homes and imposing visiting bans on everyone" (N3, Pos. 63)
